# RETRACTED: Rehman, M.U.; Rather, I.A. Myricetin Abrogates Cisplatin-Induced Oxidative Stress, Inflammatory Response, and Goblet Cell Disintegration in Colon of Wistar Rats. *Plants* 2020, *9*, 28

**DOI:** 10.3390/plants15091343

**Published:** 2026-04-28

**Authors:** Muneeb U. Rehman, Irfan A. Rather

**Affiliations:** 1 Department of Clinical Pharmacy, College of Pharmacy, King Saud University, P.O. Box-2457, Riyadh 11451, Saudi Arabia; 2 Division of Biochemistry, Faculty of Veterinary Science and Animal Husbandry, SKAUST-Kashmir, Alustang, Srinagar, J&K 190006, India; 3 Department of Biological Sciences, Faculty of Science, King Abdulaziz University (KAU), P.O. Box-80141, Jeddah 21589, Saudi Arabia

The journal retracts the article titled “Myricetin Abrogates Cisplatin-Induced Oxidative Stress, Inflammatory Response, and Goblet Cell Disintegration in Colon of Wistar Rats” [1], cited above.

Following publication, concerns were brought to the attention of the Editorial Office regarding the integrity of images presented in this article [1].

In accordance with the journal’s standard procedures, the Editorial Office and Editorial Board conducted an investigation that identified indications of inappropriate image editing and duplication between Figure 4 published in [1] and Figure 4 previously published in [2], produced by a similar authorship group and presenting different experimental conditions. Although the authors cooperated with the investigation, the explanations and supporting materials provided were not deemed sufficient by the Editorial Board to resolve the identified concerns. Consequently, the Editorial Board has lost confidence in the reliability of the reported findings and has decided to retract this publication.

This retraction was approved by the Editor-in-Chief of the journal *Plants*.

Irfan A. Rather agreed to this retraction. The remaining author did not provide a comment on this decision.
